# Assessing the potential determinants of national vitamin A supplementation among children aged 6–35 months in Ethiopia: further analysis of the 2019 Ethiopian Mini Demographic and Health Survey

**DOI:** 10.1186/s12887-022-03499-5

**Published:** 2022-07-22

**Authors:** Tadele Abate Lucha, Teklu Assefa Engida, Admassu Ketsela Mengistu

**Affiliations:** 1 Department of Neonatal Nursing, Menelik II Medical & Health Sciences College, Kotebe University of Education, Addis Ababa, Ethiopia; 2 Department of Neonatal Nursing, Menelik II Medical & Health Sciences College, Kotebe University of Education, Addis Ababa, Ethiopia; 3 Department of Pharmacy, Menelik II Medical & Health Sciences College, Kotebe University of Education, Addis Ababa, Ethiopia

**Keywords:** Vitamin a supplementation, Children, Ethiopia

## Abstract

**Background:**

Vitamin A is a nutrient that is required in a small amount for normal visual system function, growth and development, epithelia’s cellular integrity, immune function, and reproduction. Vitamin A has a significant and clinically important effect since it has been associated with a reduction in all-cause and diarrhea mortality. The aim of this study was to determine factors associated with national vitamin A supplementation among children aged 6–35 months.

**Method:**

The data for this study was extracted from the 2019 Ethiopian Mini Demographic and Health Survey. A total weighted sample of 2242 women with children aged 6–35 months was included in the study. The analysis was performed using Stata version 14.2 software. Applying sampling weight for descriptive statistics and complex sample design for inferential statistics, a manual backward stepwise elimination approach was applied. Finally, statistical significance declared at the level of *p* value < 0.05.

**Result:**

The overall coverage of vitamin A supplementation among children aged 6–35 months for the survey included was 44.4 95% CI (40.15, 48.74). In the multivariable analysis, mothers who had four or more antenatal visits [AOR = 2.02 (95% CI: 1.34, 3.04)] were two times more likely to receive vitamin A capsules for their children than mothers who had no antenatal visits. Children from middle-wealth quintiles had higher odds of receiving vitamin A capsules in comparison to children from the poorest wealth quintile [AOR = 1.77 (95% CI: 1.14, 2.73)]. Older children had higher odds of receiving vitamin A capsules than the youngest ones. Other factors that were associated with vitamin A supplementation were mode of delivery and region.

**Conclusion:**

The coverage of vitamin A supplementation in Ethiopia remains low and it is strongly associated with antenatal visit, household wealth index and age of child. Expanding maternal health services like antenatal care visits should be prioritized.

## Background

Vitamin A is a nutrient that is required in a small amount for normal visual system function, growth and development, epithelia’s cellular integrity, immune function, and reproduction [1]. Vitamin A has a significant and clinically important effect since it has been associated with a 12 and 34% reduction in all-cause and diarrhea mortality [2, 3]. Globally, the prevalence of vitamin A deficiency (VAD) related vision loss increased from 68.8 per 100,000 people in 1990 to 75.1% in 2017 [4]. Additionally, vitamin A deficiency was found to be linked to childhood stunting [5, 6].

In 2016, 64% of children in need in priority countries received two doses of vitamin A, but more than 140 million children were left behind, putting them at risk of disease and death [7].

In low-income countries, vitamin A supplementation for children aged 6–59 months is being introduced in child health programs. In many countries, however, vitamin A supplementation.

(VAS) coverage is less than 80% and 5.2 million preschool-age children have clinical vitamin A deficiency [8]. In sub-Saharan African countries, the overall coverage of vitamin A supplementation among children aged 6–59 months was 59.4% [9]. In Ethiopia, between 169,563 and 376,030 child lives have been saved as a result of vitamin A supplementation over the last 15 years 10].

So far, in Ethiopia, based on the global nutrition report, the coverage of two high-dose vitamin A supplements were 66% by 2020 [10], and at least 75% of the targeted population received at least one dose of vitamin A throughout the last 14 years (2005–2018) [11].

According to the Ethiopian demographic and health survey (EDHS), 46.8, 53, and 45% of eligible children received the supplement in the last 6 months of the survey in 2005, 2011, and 2016, respectively [12–14], but this percentage is still low when compared to the Ethiopian ministry of health’s goal of increasing the proportion of children aged 6–59 months who receive vitamin A supplementation to 95% by the end of 2020 [15].

Therefore, this study aimed to use the 2019 Ethiopian mini demographic and health survey (EMDHS) to determine factors that predict VAS in children aged 6–35 months. This may help to evaluate food and nutrition policies [16], the Ethiopian ministry of Health’s health sector transformation plan two (HSTP II), which targets to strengthen and scale up vitamin A supplementation to children aged 6–59 months by the end of 2024 [17].

## Methods and materials

### Data source

The data for this study was extracted from the 2019 EMDHS [18]. The 2019 EMDHS is the second EMDHS and the sixth DHS implemented in Ethiopia. The survey was conducted in nine regional states and two city administrations of Federal Democratic Republic of Ethiopia [18].

### Inclusion/exclusion criteria

The inclusion criteria were children aged 6–35 months and completed forms about their personal information. Hence, children aged greater than or equal to 36 months and variables that don’t contain full information like husband’s occupation (V704), husband’s education (V701) and respondent’s occupation (V716) were excluded.

### Sampling and data collection

The 2019 EMDHS sample was stratified and selected in two stages. There were nine regions and two administrative towns. Each region was stratified into urban and rural areas, yielding 21 sampling strata. In the first stage, a total of 305 enumeration areas (93 in urban areas and 212 in rural areas) were selected with probability proportional to enumeration area size based on the 2019 Ethiopian Population and Housing Census (EPHC) frame and with independent selection in each sampling stratum [18]. In the second stage of selection, a fixed number of 30 households per cluster were selected with an equal probability systematic selection from the newly created household listing. All women aged 15–49, who were either permanent residents of the selected households or visitors who slept in the household the night before the survey were eligible to be interviewed [18]. The Woman’s questionnaire was used to interview women aged 15–49. The IR file was used for analysis.

The survey interviewed 8855 women of reproductive age (age 15–49) from a national representative sample of 8663 households. Only children aged 6–35 months who lived with an eligible respondent were included in our analysis, depending on the denominator of vitamin A supplements received in the 6 months before the survey based on the Woman’s Questionnaire, resulting in a total weighted sample of 2242 women with children aged 6–35 months.

### Dependent variable

The dependent variable for this study was vitamin A supplementation in the past 6 months among children aged 6–35 months (H34 on the EMDHS data). The response was recorded by asking the question, “Whether a vitamin A dose was given?” The responses were recorded as either (yes = 1) or (no = 0).

### Independent variable

The mother’s education, maternal age, child age, household wealth index, religion, place of residence, region of residency, professional antenatal and postnatal care, place of delivery, mode of delivery, type of birth, sex of child, mother’s marital status, and parity were the primary independent variables. They were selected from the available similar studies on the subject [19–21].

### Statistical analysis

The analysis was performed using Stata version 14.2 software. Applying sampling weight for descriptive statistics and complex sample design for inferential statistics.. To accommodate for the differential probability of study participants’ being selected by region of residence, sample weights were used. Adjustments to the cluster sampling design, weights, and standard error calculation were made using “Svy” commands. Survey logistic regression was used to adjust for the complex sampling design and weights. The association between the independent variable and vitamin A supplementation was first investigated using binary logistic regression analysis. Second, a multivariate logistic regression model was used to investigate the factors linked to vitamin A supplementation (*p* value < 0.25). We ordered the covariates from most-to-least important, and manual backward stepwise elimination approach was applied [22]. With 95% confidence intervals, both unadjusted and adjusted odds ratios (OR) were presented. Finally, statistical significance was declared at the level of *p* value.

## Results

### Characteristics of the respondents

As summarized in Table 1, most of the children (72.6%) lived in rural area, most of them in Oromia (38%), Amhara (21.5%) and SNNPR (20.5%). Only 14.6% of babies had a post-natal check-up. More than one third (48.6%) of the mothers had no education. Overall, 52.9% of mothers were between the ages of 25 and 34. At the time of the survey, the majority (94.9%) said they were currently married. In terms of delivery place, 52.7% of mother gave birth in a hospital. Of these, 5.7% were delivered by caesarean section. Regarding antenatal visits, 31.7% of mothers reported having attended at least 1–3 antenatal visits during their pregnancy, while 43.6% reported having gone to more than 4 (Table 1).

**Table 1:** Background characteristics of women with children aged 6–35 months of age in Ethiopia, EMDHS 2019 (*n* = 2242)

Background characteristics	Weighted Frequency (n)	Weighted Percent (%)
**Age of child (month)**		
06–12	580	25.9
13–24	1002	44.7
25–35	660	29.4
**Child sex**		
Male	1174	52.4
Female	1068	47.6
**Region**		
Tigray	149	6.7
Afar	30	1.3
Amhara	481	21.5
Oromia	865	38.6
Somali	124	5.5
Benishangul Gumuz	27	1.2
SNNPR	460	20.5
Gambela	10	0.4
Harari	6	0.3
Addis Ababa	76	3.4
Dire Dawa	12	0.6
**Place of residence**		
Urban	615	27.4
Rural	1627	72.6
**Maternal Educational Level**		
No education	1089	48.6
Primary	858	38.3
Secondary/higher	295	13.2
**Religion**		
Orthodox	805	35.9
Protestant	662	29.5
Muslim	739	33.0
Traditional	36	1.6
**Age of Mother**		
15–24	609	27.2
25–34	1187	52.9
35–49	446	19.9
**Wealth index**		
Poorest	456	20.3
Poorer	484	21.6
Middle	444	19.8
Richer	379	16.9
Richest	479	21.4
**Currently marital status of mother**		
Married	2128	94.9
No longer living together/separated	113	5.1
**Types of birth**		
Single birth	2220	99.0
Twine	21	1.0
**Place of delivery**		
Home	1061	47.3
Health	1181	52.7
**Mode of delivery**		
Normal (vaginal)	2114	94.3
Cesarean section	127	5.7
**Antenatal care**		
No ANC visit	553	24.7
1–3	711	31.7
4+	977	43.6
**Post-natal check up**		
No	1915	85.4
Yes	327	14.6
**Parity**		
1–2	931	41.5
3–4	597	26.6
5–6	410	18.3
7+	304	13.6

### Level of vitamin a supplementation

In this study, the prevalence of vitamin A supplementation within the last 6 months in Ethiopia was observed to be 44.4 95% CI (40.15, 48.74) (Table 2). There was regional variation in child vitamin A supplementation, with Benishangul Gumuz 63%, Gambela 62%, Tigray 61%, Amhara 57%, SNNPR 35%, Afar 32% and Somali 22% (Table 2).

**Table 2:** Vitamin A supplementation among children aged 6–35 months in the regional administration of Ethiopia, EMDHS 2019 (n = 2242)

Region	Numbers of children	Percentage given vitamin A supplementation in the past six month
Tigray	149	61%
Afar	30	32%
Amhara	481	57%
Oromia	865	42%
Somali	124	22%
Benishangul Gumuz	27	63%
SNNPR	460	35%
Gambela	10	62%
Harari	6	48%
Addis Ababa	76	44%
Dire Dawa	12	56%
Total (Ethiopia)	2242	44.4%

### Vitamin a supplementation by place of residence

Among children aged 6–35 months, those in urban (49%) and rural (43%) areas received a vitamin A supplement in the 6 months before the survey (Table 3).

**Table 3:** Vitamin A supplementation among children aged 6–35 months in Ethiopia, by place of residence, EMDHS 2019 (*n =* 2242)

Place of residence	Number	Number of supplemented children	Percent
Urban	615	303	49%
Rural	1627	693	43%

### Factors associated with vitamin a supplementation

The result reveals that antenatal care, age of child and household wealth index were independently and significantly associated with vitamin A supplementation.

Mothers who had four or more ANC visits [AOR = 2.02 (95% CI: 1.34, 3.04)] were two times more likely to receive vitamin A capsules for their children, than mothers who had no ANC visits. Age of child, older children were significantly more likely to receive VAS than those aged 6–12 months (Table 4).

**Table 4:** Multivariate logistic regression analysis of factors associated with children’s receipt of a vitamin A capsule in the last six months in Ethiopia, EMDHS 2019

Variable	Full model COR at 95% CI	Reduced model AOR at 95% CI
**Post-natal check up**		
No	1	
Yes	1.79 (1.27, 2.53)	
**Antenatal care**		
No ANC visit	1	1
1–3	1.66 (1.05, 2.63)	1.28 (0.78, 2.11)
4+	2.56 (1.79, 3.67)	2.02 (1.34, 3.04) ***
**Place of delivery**		
Home	1	
Health	1.56 (1.13, 2.17)	
**Maternal Educational Level**		
No education	1	
Primary	1.13 (0.82, 1.55)	
Secondary and higher	1.48 (0.93, 2.36)	
**Place of residence**		
Urban	1.31 (0.89, 1.92)	
Rural	1	
**Region**		
Tigray	1	1
Afar	0.30 (0.14, 0.63)	0.40 (0.18, 0.86) *
Amhara	0.85 (0.42, 1.71)	0.89 (0.44, 1.80)
Oromia	0.46 (0.23, 0.94)	0.52 (0.26, 1.03)
Somali	0.17 (0.08, 0.35)	0.28 (0.13, 0.63) **
Benishangul Gumuz	1.10 (0.53, 2.25)	1.07 (0.51, 2.23)
SNNPR	0.34 (0.17, 0.65)	0.35 (0.18, 0.69) **
Gambela	1.03 (0.42, 2.47)	1.27 (0.50, 3.20)
Harari	0.57 (0.27, 1.21)	0.67 (0.30, 1.50)
Addis Ababa	0.52 (0.25, 1.04)	0.32 (0.13, 0.76) **
Dire Dawa	0.80 (0.41, 1.55)	0.76 (0.36, 1.60)
**Age of Mother**		
< 20	1	
20–34	1.29 (0.85, 1.94)	
35–49	1.63 (1.08, 2.45)	
**Mode of delivery**		
Normal (vaginal)	0.31 (0.18, 0.54)	0.40 (0.22, 0.73) **
Cesarean section	1	1
**Age of child (months)**		
05–12	1	**1**
13–24	1.77 (1.27, 2.45)	1.85 (1.29, 2.66) ***
23–35	2.07 (1.40, 3.07)	2.15 (1.44, 3.21) ***
**Wealth index**		
Poorest	1	1
Poorer	1.08 (0.73, 1.59)	0.80 (0.53, 1.20)
Middle	2.13 (1.38, 3.28)	1.77 (1.14, 2.73) **
Richer	1.24 (0.81, 1.88)	0.89 (0.55, 1.46)
Richest	1.82 (1.16, 2.83)	0.78 (0.44, 1.39)

*Significant at 0.05

**Significant at 0.01

**Significant at 0.001

In terms of household wealth index, children from the middle-wealth quintiles [AOR = 1.77 (95% CI: 1.14, 2.73)] had higher odds of receiving a vitamin A capsule in comparison to children from the poorest wealth quintile (Table 4).

## Discussion

Based on data from the Ethiopian Mini Demographic and Health Survey, this population-based study was used to assess the factors associated with VAS in children aged 6–35 months. Correspondingly, the number of antenatal visits, age of child and household wealth index were independently and significantly associated with VAS.

In this study, the number of antenatal care visits was significantly associated with VAS. Mothers who had four or more ANC visits were two times more likely to give vitamin A capsules by their health worker to their children than mothers who had no ANC visits. The finding was supported by a study in Nigeria [23] and national studies [24–26]. This could be explained by the fact that while attending antenatal care during pregnancy, the health professionals encourage, educate, and counsel the mothers about the benefits of vitamin A supplementation and the consequences of VAD. This information about VAS and immunizations, can increase the chance of receiving a vitamin A capsule for their children [26, 27]. Our findings show that the ANC was significantly associated with the VAS, so those mothers who come for the service are an important time for communicating with them and positively impacting their children’s health. The finding of our study, also supported by another study [28], indicates that children whose mothers had no ANC follow-up were more likely to suffer from VAD.

Our study also discovered that the household wealth index was significantly associated to VAS. Children from middle-wealth quintiles had higher odds of receiving a vitamin A capsule in comparison to children from the poorest wealth quintile. This finding is supported by studies conducted in Bangladesh [21] and Nigeria [29]. The possible explanation might be better household wealth standing may improve uptake of the supplement through advancing access to health information and mitigating economic barriers for seeking health care. Different studies report Socioeconomic inequality is a major determinant for maternal health service utilization [30–32], which indicates maternal health service utilization is low in the poor. One study showed children from the poorest family are more vitamin A deficient compared to those in richer family [33]. These finding shows that the wealth of a family has far reaching consequences on the health of the future born children.

Age of the child was found to be significantly associated with VAS, children with older age were more likely to have VAS as compared to younger children, a finding which is consistent with studies done in Bangladesh [20, 21] and Nigeria [29]. This could be due to the fact that older children have a longer window for receiving VAS, whereas younger children may have been ineligible (age less than 6 months) for VAS in the 6 months prior to the survey.

In this study modes of delivery and region were also significantly associated with vitamin A supplementation. Children from the Afar, Somali, SNNPR, and Addis Ababa regions were less likely to receive VAS than children from Tigray regions. This finding is supported by similar studies done in Ethiopia [24], especially in the regions of Afar and Somalia [30]. The possible explanation in the pastoralist regions (Afar and Somali) and SNNPR might be a shortage of healthcare providers and inequality in maternal healthcare service [31]. Therefore, the regional health bureau, ministry of health, and policymakers should give attention to the above-mentioned regions to enhance VAS. In the case of Addis Ababa, we suggest further analysis.

We used data from the 2019 EMDHS, which is a national survey, which is one of the study’s strengths. The key drawback is that the current study was prone to recall bias. The study’s other limitations were that it overlooked certain crucial potential factors like birth order, mother’s occupation, and father’s characteristics that could influence practice due to incomplete data.

## Conclusion

In our study, variables like antenatal visits, age of child, and household wealth index were independently and significantly associated with vitamin A supplementation, and variables like mode of delivery and region were also significantly associated with VAS.

Therefore, the authors recommend increasing awareness about VAS, especially for mothers with no antenatal follow-up and those from the lower wealth quintiles, which could be done utilizing the health extension workforce. We also recommend the regional health authorities to expand maternal health services like ANC.

## Data Availability

The survey datasets used in this study was based on publicly available dataset that is freely available online with no participant’s identity from http://www.dhsprogram.com/data/available-datasets.cfm. The minimal data used for this study are available from the corresponding author on reasonable request.

## Abbreviations

AOR: Adjusted odds ratio
COR: Crude odds ratio
CI: Confidence interval
DHS: Demographic and Health Survey
EMDHS: Ethiopia Mini Demographic and Health Survey
EPHC: Ethiopian Population and Housing Census
SNNP: Southern Nations Nationalities and Peoples
VAS: vitamin A supplementation
VAD: vitamin A deficiency
